# The potential of mineral weathering of halophilic-endophytic bacteria isolated from *Suaeda salsa* and *Spartina anglica*

**DOI:** 10.1007/s00203-022-03129-9

**Published:** 2022-08-17

**Authors:** Jun Xi, Kaiqiang Qian, Lidong Shan, Jing Huang, Yanan Yan

**Affiliations:** 1 College of Life Science, Bengbu Medical College, Bengbu, 233030 People’s Republic of China; 2 College of Life Science, Nanjing Agricultural University, Nanjing, 210095 People’s Republic of China

**Keywords:** Halophilic-endophytic bacteria, Bacterial community, Mineral weathering

## Abstract

Bacteria have the abilities of salt tolerant, mineral weathering and plant growth promoting can promote the growth of plants in saline lands. However, few reports of the mineral weathering capacity of halophilic-endophytic bacteria, raising the question of whether the halophilic-endophytic weathering bacteria are fundamentally distinct from those in plants communities. In this study, we isolated and characterized halophilic bacterial strains from the roots and leaves of *Suaeda salsa* and *Spartina anglica* with respect to their mineral weathering pattern, role in the promoting plant growth, community structure, and their changes in these two plants. Using improved Gibbson medium, we obtained 156 halophilic bacterial strains, among which 92 and 64 strains were isolated from the *S. salsa* and *S. anglica* samples, respectively. The rock weathering patterns of the isolates were characterized using batch cultures that measure the quantity of Si, Al, K, and Fe released from crystal biotite under aerobic conditions. Significantly, the biomass and capacity of the mineral weathering of the halophilic-endophytic bacteria were different in the plants. The abundance of the halophilic-endophytic bacterials in the *Suaeda salsa* was significantly greater than *Spartina anglica*, whereas the mineral weathering bacterial in the *Suaeda salsa* was similar to the *Spartina anglica*. Furthermore, the proportion of plant growth-promoting bacteria in the *Suaeda salsa* was higher than *Spartina anglica*. Phylogenetic analyses show that the weathered minerals were inhabited by specific functional groups of bacteria (*Halomonas*, *Acinetobacter*, *Burkholderia*, *Alcaligenes*, *Sphingobium*, *Arthrobacter*, *Chryseobacterium*, *Paenibacillus*, *Microbacterium*, *Ensifer*, *Ralstonia* and *Enterobacter*) that contribute to the mineral weathering. The changes in halophilic endophytes weathering communities between the two plants were attributable not only to major bacterial groups but also to a change in the minor population structure.

**Supplementary Information:**

The online version contains supplementary material available at 10.1007/s00203-022-03129-9.

## Introduction

Soil salinization is one of the most serious environmental problems in the world (Alharby et al. 2018; Jafari et al. 2018; Walter et al. 2018; Wang et al. 2018; Yuan 2015). For variety of reasons, such as vegetation destruction, water pollution, and inappropriate irrigation problem, the process of soil salinity speeds up year by year. But more and more people realized the tidal flats, which were very precious biological resources and have yet to be used and exploited. Many halophilic bacteria play an important role in salt-tolerant plants of tidal flats (Aljohny 2015). According to the bacterial growth, demand for NaCl and the optimum growth concentration of NaCl, Larsen and Vreeland divide bacteria into four categories (Larsen et al. 1987; Vreeland 1987): non-halophilic bacteria (non-halophiles), which do not need NaCl or low concentration of NaCl (< 1%) and the high concentration of NaCl will inhibit their growth; mild halophilic bacteria (slight halophiles) which need a small amount of NaCl and the optimum growth concentration of NaCl for 1–3%; moderate halophilic bacteria (moderate halophiles) which growth cannot leave the NaCl and the optimum growth concentration of NaCl for 5–10%; extremely halophilic bacteria (extreme halophiles) which are in high demand for the growth of NaCl and the optimum growth concentration of NaCl for 13–15%. The moderate halophilic bacteria and extremely halophilic bacteria belong to the extremophiles. Moderate halophilic bacteria (moderate halophiles bacteria) are paid more and more attention in recent years, the related research is increasingly extensive and detailed (Bader et al. 2018; Jafari et al. 2018; Vogt et al. 2018; Zhao et al. 2017).

Weathering of rocks plays an important role in soil formation; it represents a source of nutrients to terrestrial ecosystems and is also a major long-term sink for atmospheric CO_2_ (Baloghbrunstad et al. 2008; Hilley and Porder 2008; Uroz et al. 2009b; Wu et al. 2007). So, there has been much interest in the weathering of rocks (Hutchens et al. 2010; Oliveira et al. 2018; Wang et al. 2011). Mineral weathering is bound to biological processes associated with microorganisms, which influence various mineral transformation reactions (Frey et al. 2010; Calvaruso et al. 2006). Field observations and laboratory experiments demonstrate that microbes can accelerate mineral weathering reactions by producing organic and inorganic acids, producing metal-complexing ligands, changing redox conditions, or mediating the formation of secondary mineral phases (Barker et al. 1998). To date, increasing evidences point to a significant role for microbes in mediating mineral weathering (Chen et al. 2016; Colin et al. 2017; Uroz and Oger 2017). Many mineral-associated microbial communities, including endophytic bacteria, cyanobacteria, chemoorganotrophic and chemolithotrophic bacteria, microalgae, lichens, and fungi can accelerate mineral weathering (Kim et al. 2004). Recent studies have demonstrated bacterial community composition and diversity presented in natural and extreme environment (Alexis Carteron et al. 2022; Pantucek et al. 2018; Poddar and Das 2018; Wang et al. 2022). In addition, there is a large body of knowledge concerning the distribution, community structure, and mineral weathering roles of bacterial communities in temperate forest ecosystems (Calvaruso et al. 2010; Uroz et al. 2019; Schweiger and Laliberté 2022). Furthermore, many researchers in high salt environment of inland and coastal or ocean environments halophilic archaea are discussed in detailed research. But less study for moderate halophilic-endophytic bacteria provides the elements to the plant by weathering rock.

*Suaeda salsa* and *Spartina anglica* were the main plants at salt marshes (110,000 hectares) in Yancheng, China. We hypothesized that the endophytic bacteria of roots and leaves in the process of transferring to rhizosphere microorganisms related to soil formation. In this study, we investigated endophytic bacteria of *Suaeda salsa* and *Spartina anglica* growing on the seashore with cultivation-dependent techniques. The objectives were to analyse the diversity of culturable halophilic-endophytic bacteria, to characterize the isolates and to select the potential ability of plant growth-promoting bacteria which might promote plant growth and element uptake under the unfavorable environmental conditions.

## Materials and methods

### Isolation of halophilic-endophytic bacteria

Endophytic bacteria were isolated from leaves and roots of halophilic plants. Two halophilic plants were collected from the seashore located in Yancheng, China (32º59ʹ30ʺ–33º0ʹ31ʺN、120º49ʹ40ʺ–120º51ʹ4ʺE). Plant samples were washed with tap water followed by three rinses with distilled water and then separated into roots and leaves. Healthy plant tissues were sterilized by sequential immersion in 75% (v/v) ethanol for 2 min and 1% mercuric chloride for 1 min and washed three times with distilled water to remove surface sterilization agents. To confirm the surface disinfection process was successful, water from the final rinse was plated on improved Gibbson agar (ddH_2_O 1.0L, casein hydrolysate 5.0 g, Sodium Citrate 3.0 g, yeast extract powder 10.0 g, KCl 2.0 g, peptone 5.0 g, MgSO_4_·7H_2_O 20 g, NaCl 150 g, pH7.0). No contamination was found. Plant tissue materials (0.2 g) were ground in a mortar and pestle in the presence of 5 ml of sterile distilled water. Sterile quartz sand was added to the mortar to improve cell wall disruption. Serial dilutions were spread on improved Gibbson agar with 0.5–20% NaCl. The improved Gibbson medium was found to be most suitable for the isolation of halophilic bacteria in preliminary experiments. To prevent the growth of endophytic fungi, the media were supplemented with 10-mg fungicidin (USP, Amresco, USA) L1 after autoclaving. Plates were incubated for 72 h at 28 ℃. Halophilic-endophytic bacteria colonies were picked randomly and purified by streaking three to four times on the same media. 258 endophytic bacterial isolates growing well on subculturing were finally selected and stored at slants for further study.

### Extraction of DNA from bacterial isolates, PCR amplification

DNA was extracted from each isolate after growth until late exponential phase in improved Gibbson medium using the standard lysozyme-SDS-Pronase protocol (Sambrook et al. 1989). 16S rRNA was amplified by PCR with a thermocycler (PTC200, BIO-RAD) using an initial denaturing step of 5 min at 94 ℃ followed by 30 cycles of 45 s at 94, 1 min of annealing at 55 ℃, and 90 s extension at 72 ℃, and final polymerization step of 72 ℃ for 10 min. Each reaction mixture (50 μl) contained 2.5 U of Taq DNA polymerase (Takara), 5 μl of 10 reaction buffer, 1.5 mM MgCl_2_, 10 pmol of universal bacterial 16S rRNA primers (27f, 5′-AGA GTT TGA TCC TGG CTC AG; and 1492r, 5′- TAC GGC TAC CTT GTT ACG ACT T), 0.5 μl of template DNA, 200 μM each dNTPs (Takara). Purified PCR products from the 16S rRNA genes of all the pure cultured bacteria were sequenced on an ABI 3730 × 1automated sequencer (Invitrogen) combined with a Sequencing Kit (BigDye Terminator) and the primers set 27f and 1492r as well as M13-47 and RV-M. The resulting nucleotide sequences were blasted using the National Centre for Biotechnology Information (NCBI) database to obtain the closest species match. The phylogenetic affiliation was verified using the RDP classifier (Wang et al. 2007). The nucleotide sequences determined in this study have been deposited in the NCBI database.

### IAA (Indoleacetic acid), ACC deaminase (Aminocyclopropane-1-carboxylate deaminase) and Siderophore production

The production of IAA by the tested endophytic bacteria was determined according to the methods of Sheng et al. (Sheng et al. 2008). The production of siderophores by the bacteria was determined according to the chrome azurol-S (CAS) analytical method (Manjanatha et al. 1992). Cells and supernatants were separated by centrifugation at 9000 *g* for 10 min, and 1.0 ml of supernatant was mixed with 1.0 ml of CAS Assay Solution (Manjanatha et al. 1992). A control group was prepared by mixing 1.0 ml of the CAS Assay Solution with 1.0 ml of the noninoculated medium used for culturing bacterial strains. The absorbance at 630 nm was measured 1 h after mixing, and the values were compared with the optical density (O.D.) of the reference (Manjanatha et al. 1992). To evaluate the ACC deaminase production of the endophytic strains, strains were grown in test tubes containing 5 ml of liquid SMN medium (Belimov et al. 2005) for 24 h at 28℃ and harvested by centrifugation at 9000 *g* in 10 min at room temperature. Cell pellets were washed twice with sterile distilled water and resuspended in 1 ml of SM medium (Belimov et al. 2005). The bacteria were grown on SM medium supplemented with 3-mM ACC as the sole N source at 30 ℃ for 72 h at 150 rpm. To prepare a stock solution, ACC was dissolved in sterile distilled water and filtered through a 0.25-μm pore size membrane and stored in sterile tubes at 20 ℃. The inoculated SM medium was used as control. The bacterial growth was monitored after 72 h by measuring the O.D. at 600 nm.

### Mineral weathering experiment

We followed the methods of Sun et al. (2010). K and Fe-limited liquid medium (KFM) (containing 1% sucrose, 0.1% (NH_4_)_2_SO_4_, 0.05% Na_2_HPO_4_, 0.05% MgSO_4_, 0.01% NaCl, 0.02% yeast extract, 0.2% biotite powders, pH 7.2) was used to test mineral weathering potentials of the isolates. Unweathered crystal biotite was crushed in a jaw crusher ground and passed through sieves having a 0.149-mm mesh size. The rock powders were ultrasonically cleaned in deionized water to remove fine particles and were leached with 0.1 N HCl to remove exchangeable bases, washed with distilled water until the supernatant became clear. The elemental composition of the rock is as follows: SiO_2_ 39.99%, Al_2_O_3_ 18.98%, K_2_O 9.12%, Na_2_O 0.28%, Fe_2_O_3_ 14.75%, CaO 0.07%, and MgO 0.24%. To prepare the inoculum, bacterial strains were initially grown in sterilized improved Gibbson medium (sterilized at 121 °C for 30 min) at 28 °C for 18–20 h in a rotary shaker at 150 rpm, and harvested by centrifugation at 3000 × *g* for 10 min. Inoculum was washed two times in sterile distilled water, and cell pellets were then resuspended in saline solution (0.85% NaCl) to a final concentration of 10^8^ cells mL^−1^ before the dissolution experiments were started. Triplicate 250-mL polycarbonate Erlenmeyer culture flasks with vented caps (0.3 µm PTFE membranes) containing 50 mL of sterilized KFM were each inoculated with 2.5 mL of a bacterial suspension (Inoculum). The flasks were incubated at 28 °C on a rotary shaker at 150 rpm for 7 days. Controls with rock but no bacterial cells to monitor the range of abiotic dissolution were treated in the same manner. The weathering of the rock in the presence of bacteria was monitored at 7 days of incubation. Samples for chemical analysis were then filtered through a 5-μm Millipore filter (to retain the rock, but not the bacterial cells); 20 mL of the filtrate from each flask were centrifuged at 10,000 rpm for 10 min to remove cells of suspension. 5 mL of the supernatant were collected for pH determination and another 5 ml of the supernatant were acidified with HNO_3_ (final concentration 2% v/v) to avoid precipitation of dissolved chemical species and analyzed for Si, Fe, and K contents by ICP-AES (Atomic Emission Spectrometer).

### Statistical analysis

One-way analysis of variance (ANOVA) and the Fisher’s Least Significant Difference test (Fisher’s LSD) (*p* < 0.05) were used to compare treatment means for pH and Si, Fe, K released from the biotite mineral by the mineral weathering bacteria with the uninoculation control, the statistical analyses were carried out using SAS 8.2 (Statistical Analysis System, USA).

## Results

### Bacterial counts and isolation of halophilic bacteria

The culturable bacterial counts (log CFU g^−1^ fresh samples) of the plant samples revealed an appreciable tendency to change with different parts. More bacterial counts were obtained from the *S. salsa* samples than from the *S. anglica* samples. Endophytic bacterial counts of the two plant species ranged from 1.43 × 10^3^ to 3.76 × 10^3^ cfu g^−1^ fresh weight (Table 1). It was very noticeable that the counts of halophilic-endophytic bacteria from roots and leaves of *S. salsa* attained 10^3^ cfu g^−1^ fresh weight, with 1.33 and 2.43 × 10^3^ cfu g^−1^ fresh weight, respectively. The ratio of halophilic-endophytic bacteria to total endophytic bacteria for *S. anglica* was 100%, while the ratio for *S. salsa* was 91.7%, which indicated that halophilic bacteria were the dominant community in endophytic bacteria.

**Table 1:** The number of total and halophilic bacteria ratio

Samples	Total bacterial counts (10^3^ cfu g^−1^)^a^	halophilic bacteria ratio(%)^b^			
		5% NaCl	10% NaCl	15% NaCl	20% NaCl
*Suaeda salsa*					
Root	1.33 ± 0.15	91.7	91.7	91.7	25
Leaf	2.43 ± 0.58	100	58.3	54.2	20.1
*Spartina anglica*					
Root	0.53 ± 0.11	100	64.3	64.3	42.9
Leaf	0.9 ± 0.10	100	42.9	35.7	28.6

^a^Average of the cfu from three repetitive platings with improved Gibbson medium

^b^Average of the ratio of halophilic bacteria to total endophytic bacteria from three repetitive platings with improved Gibbson medium of different Salinity

### Isolation and phylogenetic analysis of the mineral weathering halophilic bacteria.

**Fig. 1:**
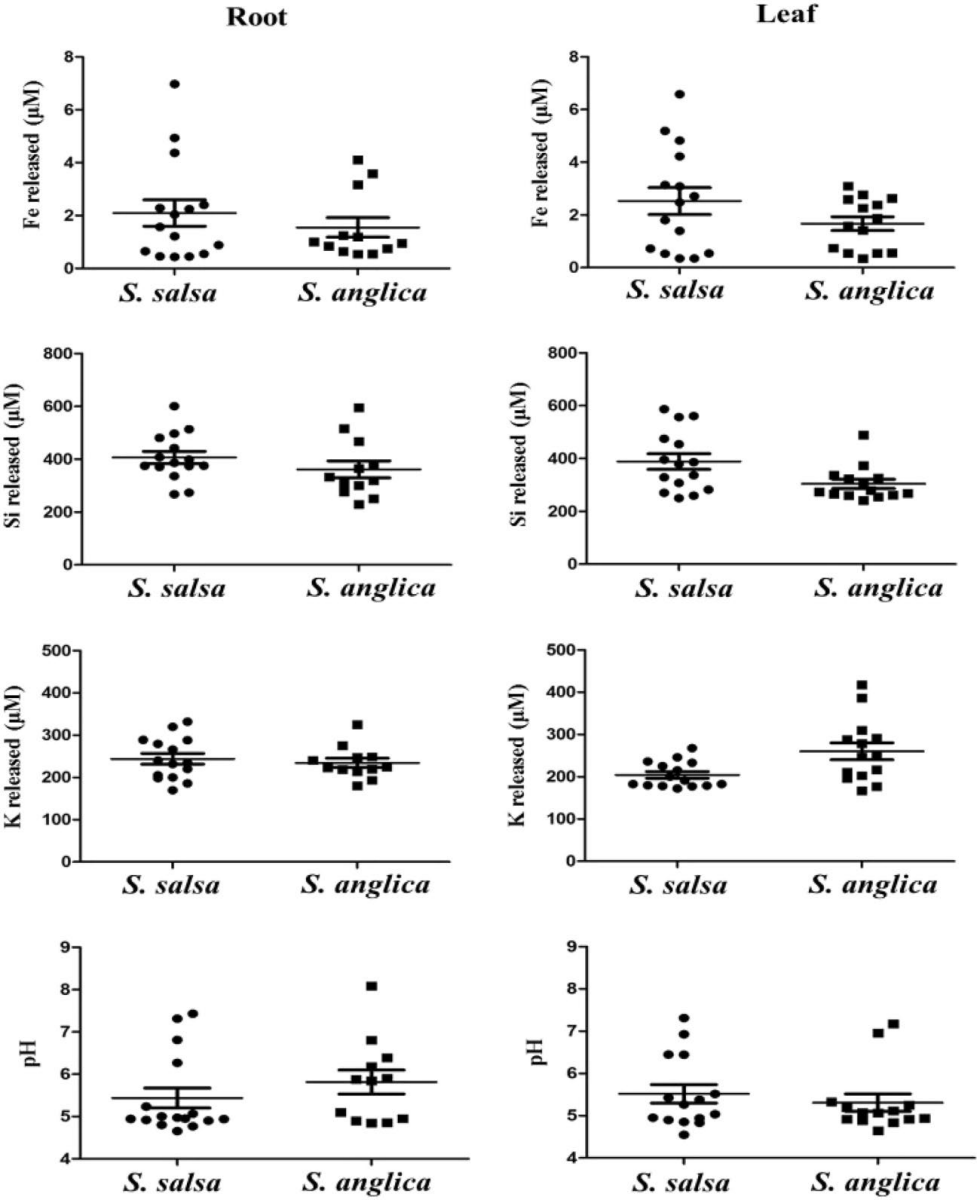
Changes in element (Fe, Si, K) release into the culture medium and changes in its pH during the weathering of biotite in the presence of mineral weathering bacteria at KFM liquid medium

Using agar plates, we obtained 156 halophilic bacterial strains, among which 92 and 64 strains were isolated from the *S. salsa* and *S. anglica* samples, respectively. In the mineral weathering experiment, the releases of major structural elements, dissolved Fe, Si and K in biotite, were used as an overall indicator of mineral weathering (Table S1). The average element releases by the bacteria isolated from *Suaeda salsa* were significantly higher than that from *S. anglica*. The K release by the isolates from leaves of *S. salsa* was lower than *S. anglica* (Fig. 1).

Based on the mineral weathering experiment, 32.6% (30/92) and 40.6% (26/64) of the strains from the *S. salsa* and *S. anglica*, respectively, were found to have the capacity to weather biotite (Fig. 1). Among the 30 mineral weathering isolates from the *S. salsa*, 15 and 15 bacterial strains were obtained from the roots and leaves, respectively; among the 26 mineral weathering bacteria from the *S. anglica*, 12 and 14 bacteria were obtained from the roots and leaves, respectively.

**Fig. 2:**
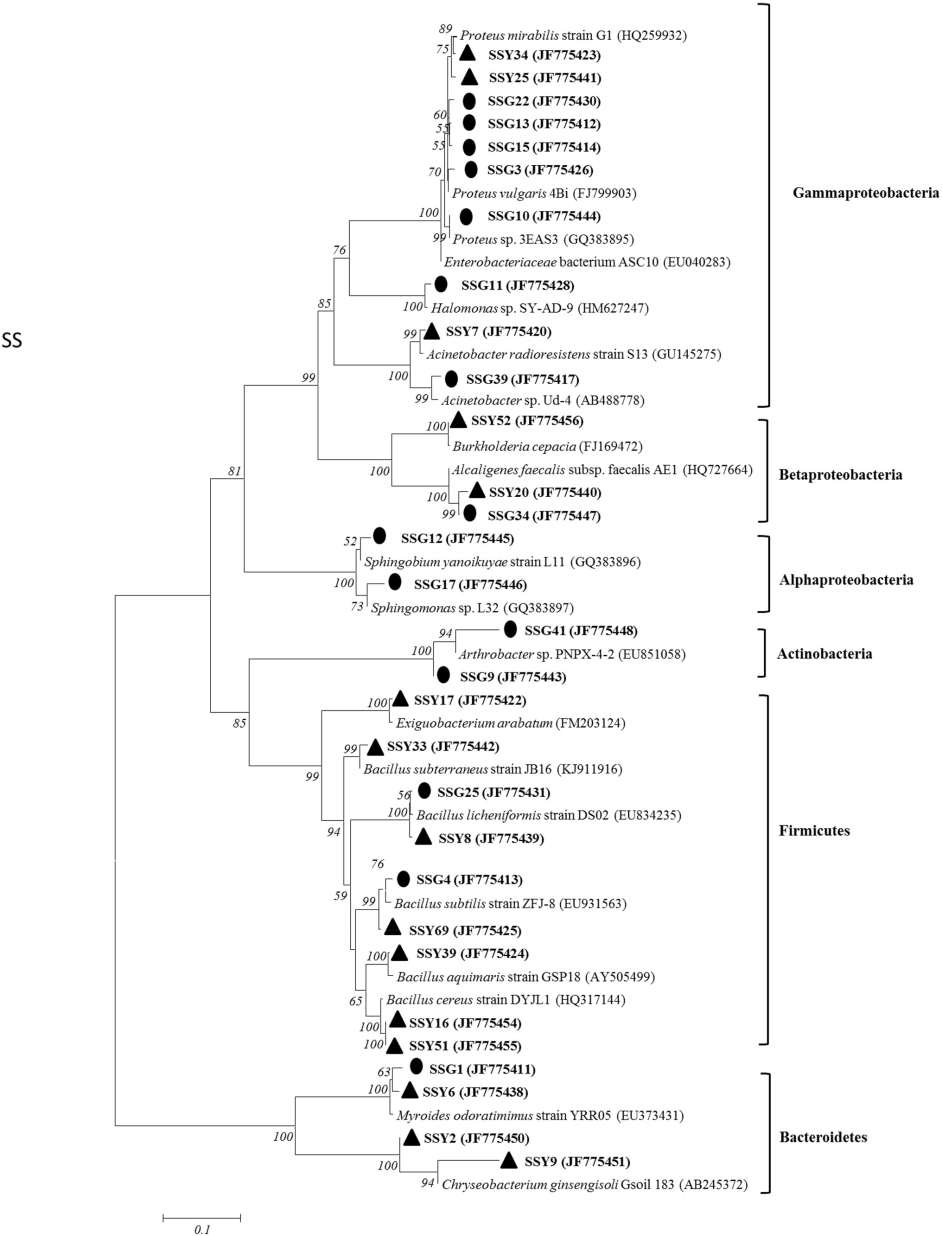
Neighbor-joining phylogenetic tree of partial 16S rRNA sequences of mineral weathering halophilic bacteria from *S. salsa*. Strains marked ● were isolated from roots samples. Strains marked ▲ were isolated from leaves samples. Bootstrap values larger than 50% (after 1000 resampling) are indicated on the branches. The scale bar represents 0.1 substitutions per nucleotide position. GenBank numbers are given in parentheses

**Fig. 3:**
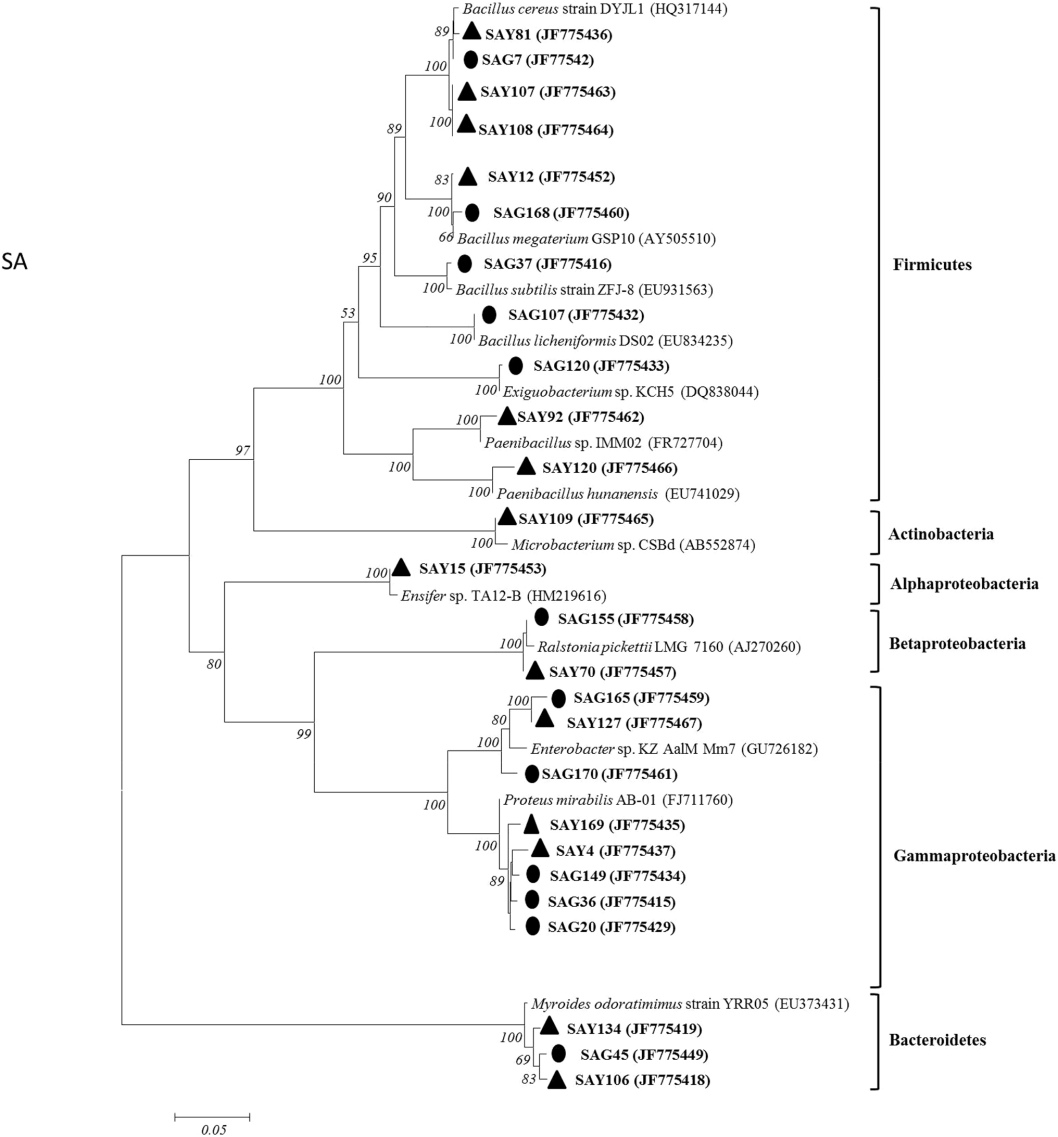
Neighbor-joining phylogenetic tree of partial 16S rRNA sequences of mineral weathering halophilic bacteria from *S. anglica*. Strains marked ● were isolated from roots samples. Strains marked ▲ were isolated from leaves samples. Bootstrap values larger than 50% (after 1000 resampling) are indicated on the branches. The scale bar represents 0.05 substitutions per nucleotide position. GenBank numbers are given in parentheses

Sequencing of the 16S rRNA and phylogenetic analysis showed that the mineral weathering bacteria were affiliated with 11 and 9 genera in the *S. salsa* and *S. anglica*, respectively (Figs. 2 and 3). Sixteen different bacterial genera were obtained from the two plants. Among the mineral weathering bacterial genera *Halomonas*, *Acinetobacter*, *Burkholderia*, *Alcaligenes*, *Sphingobium*, *Arthrobacter* and *Chryseobacterium* were specific to the *S. salsa*, while *Paenibacillus*, *Microbacterium*, *Ensifer*, *Ralstonia* and *Enterobacter* were specific to the *S. anglica*. The most frequently isolated mineral weathering bacteria from the *S. salsa* belonged to *Bacillus* (26.7%) and *Proteus* (23.3%) species. Members of *Halomonas*, *Sphingobium* and *Arthrobacter* with mineral weathering potential were specific to the roots, while members of *Burkholderia*, *Exiguobacterium* and *Chryseobacterium* with mineral weathering potential were only present in leaves. Notably, *Proteus* enriched in the roots while *Bacillus* were enriched in the leaves. The most frequently mineral weathering isolates belonged to *Bacillus* (30.8%) and *Proteus* (19.2%). *Exiguobacterium* is specific to roots and *Paenibacillus*, *Microbacterium* and *Ensifer* is specific to leaves. Enrichment of *Bacillus* and *Proteus* in the roots and leaves have little difference.

### Nucleotide sequence accession numbers

The nucleotide sequences determined in this study have been deposited in the NCBI database under accession numbers JF775411–JF775467 for halophilic-endophytic bacteria strains.

### Plant growth-promoting characteristics of the isolates.

There were more strains producing IAA from leaves (8 strains) than roots (2 strains) in the *S. anglica* (Table 2). The strains *Bacillus* (SAY108 and SAG168), *Exiguobacterium* (SAG120), *Paenibacillus* (SAY92), *Microbacterium* (SAY109), *Ensifer* (SAY15), *Enterobacter* (SAY127), *Proteus* (SAY169 and SAY4) and *Myroides* (SAY106) were able to produce IAA. Moreover, *Ralstonia* (SAG155) did not produce IAA. IAA production of the isolates form *S. salsa* (53.3%) was more than from *S. anglica* (38.5%). In the *S. salsa*, the strains *Proteus* (SSY34, SSY25, SSG13 and SSG3), *Bacillus* (SSY33, SSG25, SSG4, SSY69, SSY39, SSY16 and SSY51), *Myroides* (SSG1) and *Chryseobacterium* (SSY9) were able to produce IAA. Moreover, *Halomonas* (SSG11), *Acinetobacter* (SSY7 and SSG39), *Alcaligenes* (SSY20 and SSG34), *Sphingobium* (SSG12 and SSG17), *Exiguobacterium* (SSY17) did not produce IAA. A large fraction of the isolates (corresponding to 98.2% of the total isolates) were able to produce siderophores (Table 2), but little bacteria that produced high concentrations of siderophores (+ + +). In particular, 3 strains producing high siderophores belonged to three genera, which were *Bacillus*, *Myroides*, and *Proteus*. Among the tested isolates, only 2 strains from *S. anglica* and 4 strains from *S. salsa* belonging to six different genera (*Myroides*, *Proteus*, *Enterobacter*, *Chyseobacterium*, *Halomonas* and *Arthrobacter*) could utilize ACC as the sole N source, which indicates that they possess ACC deaminase activity.

**Table 2:** The Plant growth-promoting Characteristics of halophilic bacteria

Strains	ACC^b^ deaminasae	IAA^b^	Siderophore production^a^
SSG1	ND	+	+ +
SSG3	ND	+	+
SSG4	ND	+	+
SSG7	ND	ND	+ +
SSG9	+	+	+
SSG10	ND	ND	+
SSG11	+	ND	+
SSG12	ND	ND	+
SSG13	ND	+	+ +
SSG15	+	ND	+
SSG17	ND	ND	+
SSG20	ND	ND	+
SSG22	ND	ND	+
SSG25	ND	+	+
SSG34	ND	+	+
SAG36	ND	ND	+ +
SAG37	ND	ND	+ + +
SSG39	ND	ND	+
SSG41	ND	+	+
SAG45	+	ND	+
SSY2	ND	+	+
SAY4	ND	+	+ +
SSY6	ND	ND	+
SSY7	ND	ND	+
SSY8	ND	ND	+
SSY9	+	+	+
SAY10	ND	ND	+ +
SAY12	ND	ND	+
SAY15	ND	+	+
SSY16	ND	+	+
SSY17	ND	ND	+
SSY20	ND	ND	+
SSY25	ND	+	+ + + +
SSY33	ND	+	+
SSY34	ND	ND	+
SSY39	ND	+	+ +
SSY51	ND	ND	+
SSY52	ND	+	+
SSY69	ND	+	+ +
SAY70	ND	ND	+
SAG107	ND	ND	+ +
SAG120	ND	+	+
SAG149	ND	ND	+
SAG155	ND	ND	+
SAG165	ND	ND	+
SAG168	ND	+	+
SAY169	ND	+	+
SSG170	ND	ND	+
SAY81	ND	ND	+ +
SAY92	ND	+	ND
SAY106	ND	+	+ + +
SAY107	ND	ND	+ +
SAY108	ND	+	+
SAY109	ND	+	+
SAY120	ND	ND	+
SAY127	+	+	+
SAY134	ND	ND	+

^a^Siderophore production: values of absorbancy/absorbancy reference at 630 nm: + , 0.8–1.0; +  + , 0.6–0.8; +  +  + , 0.4–0.6; +  +  +  + , 0.2–0.4; +  +  +  +  + , 0–0.2

^b^ND: no detection

## Discussion

Many studies have been reported on the analyses of extreme environmental bacterial communities of the saline soil, marine, and microbial populations in salt lake (Aanderud et al. 2016; Adriaenssens et al. 2017; Crespo-Medina et al. 2016; Pinhassi et al. 2016; Sun et al. 2017; Wang et al. 2014), but very little known about the relationship of the mineral weathering halophilic bacterial communities in the salt-tolerant plants. In this study, 10^3^ culturable bacteria counts per gram of leaf or root were obtained in the salt-tolerant plants, suggesting that the bacteria present in the salt-tolerant plants have a range of physiological properties clearly related to their sources. Our study showed that many bacteria from salt-tolerant plants had plant growth-promoting characteristics and the ability of mineral weathering (Table 2). Although high-concentration salt exerts their osmotic pressure effects on microorganisms through various mechanisms, halophilic-endophytic bacteria can survive in these habitats and can be isolated and selected for their potential application in bioremediation of contaminated extreme environmental (Puente et al. 2009). Halophilic-endophytic bacteria can be isolated from two plant species, indicating that these endophytic bacteria populations had a marked adaptation to high-concentration salt under constant osmotic pressure for a long time. Possibly due to the higher Na^+^ concentrations in the tissues of *S. anglica*, the proportion of halophilic-endophytic bacteria (20.1–91.7%) for *S. anglica* was higher than that (28.6–100%) for *S. salsa*.

Although the plants grown in the same site, significant different in the plant parameters existed between the two plant samples (Table 2, Figs. 1, 2, 3). So the plant growth-promoting and mineral weathering bacterial communities could be compared between the two samples. The analyses of the 16S rRNA gene sequences revealed a broad diversity of the mineral weathering bacterial types in the two plant samples (Figs. 2 and 3). Although the common genera were represented in the bacterial communities of the two plant samples, some genera were found in only one plant sample, suggesting that these groups were much less represented or absent in the different plants. Similarly, distinct bacterial communities involved in mineral weathering in cacti and *Elsholtzia splendens* have been found in previous investigations (Calvaruso et al. 2010; Collignon et al. 2011; Puente et al. 2009; Uroz et al. 2007). When comparing the bacterial communities analyzed by culture dependent methods, we can find that 7 distinct bacterial genera were identified in *S. salsa* by using culture-dependent methods (Fig. 2); similarly, 5 distinct bacterial genera were also identified in *S. anglica* (Fig. 3). In addition, using culture-dependent techniques, *Bacillus* was found to be dominant in the two plant samples. In contrast, using culture-based technique, in *S. salsa* samples, the most abundant isolates belonged to *Proteus* for roots and *Bacillus* for leaves (Fig. 2). These results suggested that culturing methods will allow for understanding of the bacterial community composition and function in these complex ecosystems.

Previous studies showed that mineral weathering controls the availability of inorganic nutrients for living organisms (Uroz et al. 2009b). Uroz also showed that soil microorganisms may play an important role in soil formation and recycling of nutrients (Uroz et al. 2009a). Interestingly, it is worth noting that the proportions of mineral weathering endophytic bacteria of the different plants were higher (ranging from 32.6% to 40.6%) (Table 2), indicating that mineral weathering endophytic bacteria were the dominant community of culturable halophilic-endophytic bacteria in the two samples. The cultivation approach also revealed large variations (at the genus level) in the mineral weathering endophytic bacterial types associated with the leaves and roots (Figs. 2 and 3), indicating that the culturable bacterial communities involved in mineral weathering in the different plant samples were specialized for their environments. The ability to weather biotite minerals has been described for a range of bacterial genera belonging to *Bacillus*, *Burkholderia*, *Arthrobacter*, *Klebsiella*, *Staphylococcus*, *Acidithiobacillus*, *Sphingomonas*, *Paenibacillus*, and *Serratia* (Barker et al. 1998; Dopson et al. 2009; Honda et al. 2017; Yoshigoe et al. 2018). Although members of *Staphylococcus* and *Serratia,* which could weather biotite minerals, were not obtained in the study, our biotite dissolution experiments with strains isolated from the halophilic samples showed that isolates belonging to the genera of *Ralstonia*, *Exiguobacterium*, *Acinetobacter*, *Halomonas*, *Myroides*, *Ensifer*, *Chryseobacterium*, *Alcaligenes* could also be very effective in enhancing mineral weathering (Figs. 2, 3 and Table 2). In addition, the correlation between mineral weathering and phylogenetic divergence of bacteria showed that some bacterial groups (such as *Bacillus* and *Proteus*) which have better mineral weathering potential distribute in both plants, however, some bacterial groups which were specific to each site have been also found to be top or medium biotite solubilizers (Figs. 1, 2, 3).

Although the bacteria have the different ability to weather the biotite mineral, as evidenced by the culture solution element (Si, K, and Fe) analysis (Table 2), the enhanced rate of Si, K, and Fe release observed in the biotic systems was in agreement with other studies showing that bacteria increase the rate of silicate mineral dissolution (Chen et al. 2016; Hutchens et al. 2003; Sheng et al. 2008). It is generally assumed that element (such as Si, K, and Fe) releases from silicate minerals in the presence of bacteria were caused by proton- and/or ligand-promoted mineral weathering (Bennett et al. 1996; Buss et al. 2007). The bacteria isolated from salt-tolerant plants in different tissues had different ability to acidify the culture media (Table 2). More bacteria (15.6%) from *S. anglica* produced large pH changes (pH < 5.00 in the culture solution) than the bacteria (12.0%) from *S. salsa* in the mineral weathering experiment, indicating that the element releases from the biotite mineral might be caused by proton-promoted dissolution. However, more bacteria (6.5%) from *S. salsa* produced small pH changes (pH > 6.50 in the culture solution) than the bacteria from *S. anglica*, indicating that the element releases from the biotite mineral might be caused by ligand-promoted dissolution by bacteria from *S. salsa*. In addition, near the same number of bacteria from *S. salsa* and *S. anglica* produced medium pH changes (5.00 < pH < 6.50 in the culture solution), indicating that proton-and ligand-promoted dissolutions might be the reaction mechanism used by these bacteria for mineral weathering. Despite the difficulties associated with attempting to interpret the complex processes occurring in the natural environment based on simple laboratory experiments, and the magnitude of bacterial involvement in mineral weathering effects in natural environments is still poorly understood, it is still possible to glean information about the basic principles of bacterially mediated mineral weathering in experimental work.

Most of the commonly known salt-tolerant plants have a slow growth rate and low biomass. The plant growth-promoting bacteria could promote the growth and nutrient element uptake of plants has been reported (Madhaiyan et al. 2007; Rajkumar and Freitas 2008; Sheng and Xia 2006). The characteristics of IAA, siderophores, ACC deaminase produced by bacteria may have the potential for the promotion of plant growth and nutrient element uptake (Dellamico et al. 2008; Jiang et al. 2008; Ma et al. 2009; Sheng et al. 2008). A large portion of mineral weathering halophilic bacteria possessed the plant growth-promoting characteristics such as siderophore and IAA production and some strains showed ACC deaminase activity (Table 2). The plant growth-promoting endophytic bacteria not only promoted the growth of plant after colonized in vivo, but also defensed the disease and improved the nutrient element of plants. Therefore, the colonization of the plant growth-promoting endophytic bacteria in plant and the mechanisms of bacteria-plant interactions were worthy investigated.

This study provides the first insight into the bacterial communities occupying salt-tolerant plants at salt marshes in Yancheng, China. We demonstrated that the different salt-tolerant plants harbour highly diverse and distinct bacterial communities. Bacterial mineral weathering in laboratory-based experiments showed that strains isolated from salt-tolerant plants samples could significantly increase the release of Si, K and Fe from the biotite compared to abiotic controls. In addition, except for the reported bacterial groups which have the ability to weather biotite minerals, salt-tolerant plants may be also inhabited by specific groups of bacteria involved in mineral weathering processes. These findings have important implications for an improved understanding of the different indigenous bacterial communities in these salt-tolerant plants and their relevance to the possible effect on salt-tolerant plants growth.

## Supplementary Information

Below is the link to the electronic supplementary material.

Supplementary file1 (DOC 104 KB)

## Data Availability

The data used to support the findings of this study are available from the corresponding author upon request.

## References

[CR1] Aanderud ZT, Vert JC, Lennon JT, Magnusson TW, Breakwell DP, Harker AR (2016). Bacterial dormancy is more prevalent in freshwater than hypersaline lakes. Front Microbiol.

[CR2] Adriaenssens EM, Kramer R, Van Goethem MW, Makhalanyane TP, Hogg I, Cowan DA (2017). Environmental drivers of viral community composition in Antarctic soils identified by viromics. Microbiome.

[CR3] Alharby HF, Colmer TD, Barrett-Lennard EG (2018). Salinization of the soil solution decreases the further accumulation of salt in the root zone of the halophyte *Atriplex nummularia* Lindl. growing above shallow saline groundwater. Plant Cell Environ.

[CR4] Aljohny B (2015). Halophilic bacterium–a review of new studies. Biosci Biotechnol Res Asia.

[CR5] Bader M, Muller K, Foerstendorf H, Schmidt M, Simmons K, Swanson JS, Reed DT, Stumpf T, Cherkouk A (2018). Comparative analysis of uranium bioassociation with halophilic bacteria and archaea. PLoS ONE.

[CR6] Baloghbrunstad Z, Keller CK, Dickinson JT, Stevens F, Li CY, Bormann BT (2008). Biotite weathering and nutrient uptake by ectomycorrhizal fungus, *Suillus tomentosus*, in liquid-culture experiments. Geochim Cosmochim Acta.

[CR7] Barker WW, Welch SA, Chu S, Banfield JF (1998). Experimental observations of the effects of bacteria on aluminosilicate weathering. Am Miner.

[CR8] Belimov AA, Hontzeas N, Safronova VI, Demchinskaya SV, Piluzza G, a. M., Bullitta, S. M. & Glick, B. R.  (2005). Cadmium-tolerant plant growth-promoting bacteria associated with the roots of Indian mustard (*Brassica juncea* L. Czern.). Soil Biol Biochem.

[CR9] Bennett PC, Hiebert FK, Choi WJ (1996). Microbial colonization and weathering of silicates in a petroleum-contaminated groundwater. Chem Geol.

[CR10] Buss HL, Luttge A, Brantley SL (2007). Etch pit formation on iron silicate surfaces during siderophore-promoted dissolution. Chem Geol.

[CR11] Calvaruso C, Turpault M, Freyklett P (2006). Root-associated bacteria contribute to mineral weathering and to mineral nutrition in trees: a budgeting analysis. Appl Environ Microbiol.

[CR12] Calvaruso C, Turpault M, Leclerc E, Ranger J, Garbaye J, Uroz S, Freyklett P (2010). Influence of forest trees on the distribution of mineral weathering-associated bacterial communities of the *Scleroderma citrinum* mycorrhizosphere. Appl Environ Microbiol.

[CR13] Carteron A, Vellend M, Laliberté E (2022). Mycorrhizal dominance reduces local tree species diversity across US forests. Nat Ecol Evol.

[CR14] Chen W, Luo L, He L, Wang Q, Sheng X (2016). Distinct mineral weathering behaviors of the novel mineral-weathering strains Rhizobium yantingense H66 and Rhizobium etli CFN42. Appl Environ Microbiol.

[CR15] Colin Y, Nicolitch O, Van Nostrand JD, Zhou JZ, Turpault MP, Uroz S (2017). Taxonomic and functional shifts in the beech rhizosphere microbiome across a natural soil toposequence. Sci Rep.

[CR16] Collignon C, Uroz S, Turpault M, Freyklett P (2011). Seasons differently impact the structure of mineral weathering bacterial communities in beech and spruce stands. Soil Biol Biochem.

[CR17] Crespo-Medina M, Bowles MW, Samarkin VA, Hunter KS, Joye SB (2016). Microbial diversity and activity in seafloor brine lake sediments (Alaminos Canyon block 601, Gulf of Mexico). Geobiology.

[CR18] Dellamico E, Cavalca L, Andreoni V (2008). Improvement of Brassica napus growth under cadmium stress by cadmium-resistant rhizobacteria. Soil Biol Biochem.

[CR19] Dopson M, Lovgren L, Bostrom D (2009). Silicate mineral dissolution in the presence of acidophilic microorganisms: implications for heap bioleaching. Hydrometallurgy.

[CR20] Frey B, Rieder SR, Brunner I, Plotze M, Koetzsch S, Lapanje A, Brandl H, Furrer G (2010). Weathering-associated bacteria from the Damma glacier forefield: physiological capabilities and impact on granite dissolution. Appl Environ Microbiol.

[CR21] Hilley GE, Porder S (2008). A framework for predicting global silicate weathering and CO_2_ drawdown rates over geologic time-scales. Proc Natl Acad Sci USA.

[CR22] Honda M, Shimoyama I, Kogure T, Baba Y, Suzuki S, Yaita T (2017). Proposed cesium-free mineralization method for soil decontamination: demonstration of cesium removal from weathered biotite. ACS Omega.

[CR23] Hutchens E, Valsamijones E, Mceldowney S, Gaze W, Mclean J (2003). The role of heterotrophic bacteria in feldspar dissolution—an experimental approach. Mineral Mag.

[CR24] Hutchens E, Gleeson DB, Mcdermott F, Mirandacasoluengo R, Clipson N (2010). Meter-scale diversity of microbial communities on a weathered pegmatite granite outcrop in the Wicklow Mountains, Ireland; evidence for mineral induced selection?. Geomicrobiol J.

[CR25] Jafari S, Aghaei SS, Afifi-Sabet H, Shams-Ghahfarokhi M, Jahanshiri Z, Gholami-Shabani M, Shafiei-Darabi S, Razzaghi-Abyaneh M (2018). Exploration, antifungal and antiaflatoxigenic activity of halophilic bacteria communities from saline soils of Howze-Soltan playa in Iran. Extremophiles.

[CR26] Jiang C, Sheng X, Qian M, Wang Q (2008). Isolation and characterization of a heavy metal-resistant *Burkholderi*a sp. from heavy metal-contaminated paddy field soil and its potential in promoting plant growth and heavy metal accumulation in metal-polluted soil. Chemosphere.

[CR27] Kim J, Dong H, Seabaugh J, Newell SW, Eberl DD (2004). Role of microbes in the smectite-to-illite reaction. Science.

[CR28] Larsen PI, Sydnes LK, Landfald B, Strom AR (1987). Osmoregulation in *Escherichia* coli by accumulation of organic osmolytes: betaines, glutamic acid, and trehalose. Arch Microbiol.

[CR29] Ma Y, Rajkumar M, Freitas H (2009). Inoculation of plant growth promoting bacterium *Achromobacter xylosoxidans* strain Ax10 for the improvement of copper phytoextraction by Brassica juncea. J Environ Manage.

[CR30] Madhaiyan M, Poonguzhali S, Sa T (2007). Metal tolerating methylotrophic bacteria reduces nickel and cadmium toxicity and promotes plant growth of tomato (*Lycopersicon esculentum* L.). Chemosphere.

[CR31] Manjanatha MG, Loynachan TE, Atherly AG (1992). Tn5 mutagenesis of Chinese Rhizobium fredii for siderophore overproduction. Soil Biol Biochem.

[CR32] Oliveira DP, Sartor LR, Souza VS, Correa MM, Romero RE, Andrade GRP, Ferreira TO (2018). Weathering and clay formation in semi-arid calcareous soils from Northeastern Brazil. CATENA.

[CR33] Pantucek R, Sedlacek I, Indrakova A, Vrbovska V, Maslanova I, Kovarovic V, Svec P, Kralova S, Kristofova L, Keklakova J, Petras P, Doskar J (2018). *Staphylococcus edaphicus* sp nov., isolated in antarctica harbors the mecC gene and genomic islands with a suspected role in adaptation to extreme environments. Appl Environ Microbiol.

[CR34] Pinhassi J, Delong EF, Beja O, Gonzalez JM, Pedros-Alio C (2016). Marine bacterial and archaeal ion-pumping rhodopsins: genetic diversity, physiology, and ecology. Microbiol Mol Biol Rev.

[CR35] Poddar A, Das SK (2018). Microbiological studies of hot springs in India: a review. Arch Microbiol.

[CR36] Puente ME, Li CY, Bashan Y (2009). Rock-degrading endophytic bacteria in cacti. Environ Exp Bot.

[CR37] Rajkumar M, Freitas H (2008). Effects of inoculation of plant-growth promoting bacteria on Ni uptake by Indian mustard. Biores Technol.

[CR60] Sambrook J, Fritsch EF, Maniatis T (1989). Molecular Cloning: A Laboratory Manual.

[CR38] Schweiger AK, Laliberté E (2022). Plant beta-diversity across biomes captured by imaging spectroscopy. Nat Commun.

[CR39] Sheng X, Xia J (2006). Improvement of rape (Brassica napus) plant growth and cadmium uptake by cadmium-resistant bacteria. Chemosphere.

[CR40] Sheng X, Xia J, Jiang C, He L, Qian M (2008). Characterization of heavy metal-resistant endophytic bacteria from rape (*Brassica napus*) roots and their potential in promoting the growth and lead accumulation of rape. Environ Pollut.

[CR42] Sun L-N, Zhang Y-F, He L-Y, Chen Z-J, Wang Q-Y, Qian M, Sheng X-F (2010). Genetic diversity and characterization of heavy metal-resistant-endophytic bacteria from two copper-tolerant plant species on copper mine wasteland. Biores Technol.

[CR43] Sun S, Li S, Avera BN, Strahm BD, Badgley BD (2017). Soil bacterial and fungal communities show distinct recovery patterns during forest ecosystem restoration. Appl Environ Microbiol.

[CR44] Uroz S, Oger P (2017). *Caballeronia mineralivorans* sp. nov., isolated from oak-Scleroderma citrinum mycorrhizosphere. Syst Appl Microbiol.

[CR61] Uroz S, Calvaruso C, Turpault MP, Pierrat JC, Mustin C, Frey-Klett P (2007). Effect of the mycorrhizosphere on the genotypic and metabolic diversity of the bacterial communities involved in mineral weathering in a forest soil. Appl Environ Microbiol.

[CR45] Uroz S, Calvaruso C, Turpault M, Freyklett P (2009). Mineral weathering by bacteria: ecology, actors and mechanisms. Trends Microbiol.

[CR46] Uroz S, Calvaruso C, Turpault M, Sarniguet A, De Boer WF, Leveau JHJ, Freyklett P (2009). Efficient mineral weathering is a distinctive functional trait of the bacterial genus Collimonas. Soil Biol Biochem.

[CR47] Uroz S, Calvaruso C, Turpault M, Pierrat JC, Mustin C, Freyklett P (2019). Plant symbionts are engineers of the plant-associated microbiome. Trends Plant Sci.

[CR48] Vogt JC, Abed R, Albach DC, Palinska KA (2018). Bacterial and archaeal diversity in hypersaline cyanobacterial mats along a transect in the intertidal flats of the Sultanate of Oman. Microb Ecol.

[CR49] Vreeland RH (1987). Mechanisms of halotolerance in microorganisms. Crit Rev Microbiol.

[CR50] Walter J, Luck E, Bauriegel A, Facklam M, Zeitz J (2018). Seasonal dynamics of soil salinity in peatlands: a geophysical approach. Geoderma.

[CR51] Wang Q, Garrity GM, Tiedje JM, Cole JR (2007). Naïve Bayesian classifier for rapid assignment of rRNA sequences into the new bacterial taxonomy. Appl Environ Microbiol.

[CR52] Wang Q, Ma G, He L, Sheng X (2011). Characterization of bacterial community inhabiting the surfaces of weathered bricks of Nanjing Ming city walls. Sci Total Environ.

[CR53] Wang JL, Wang F, Chu LM, Wang H, Zhong ZP, Liu ZP, Gao JY, Duan HR (2014). High genetic diversity and novelty in eukaryotic plankton assemblages inhabiting saline lakes in the Qaidam Basin. PLoS ONE.

[CR54] Wang XP, Zhang F, Ding JL, Kung HT, Latif A, Johnson VC (2018). Estimation of soil salt content (SSC) in the Ebinur Lake Wetland National Nature Reserve (ELWNNR), Northwest China, based on a Bootstrap-BP neural network model and optimal spectral indices. Sci Total Environ.

[CR59] Wang B (2022). Even short-term revegetation complicates soil food webs and strengthens their links with ecosystem functions.. J Appl Ecol.

[CR55] Wu L, Jacobson AD, Chen HC, Hausner M (2007). Characterization of elemental release during microbe–granite interactions at T = 28 ℃. Geochim Cosmochim Acta.

[CR56] Yoshigoe A, Shiwaku H, Kobayashi T, Shimoyama I, Matsumura D, Tsuji T, Nishihata Y, Kogure T, Ohkochi T, Yasui A, Yaita T (2018). Nanoscale spatial analysis of clay minerals containing cesium by synchrotron radiation photoemission electron microscopy. Appl Phys Lett.

[CR57] Yuan L (2015). Development of super hybrid rice for food security in China. Engineering.

[CR58] Zhao B, Lu W, Zhang S, Liu K, Yan Y, Li J (2017). Reclassification of *Bacillus saliphilus* as *Alkalicoccus saliphilus* gen. nov., comb. nov., and description of *Alkalicoccus halolimnae* sp nov., a moderately halophilic bacterium isolated from a salt lake. Int J Syst Evol Microbiol.

