# Supplementary material for: The potential of mineral weathering of halophilic-endophytic bacteria isolated from Suaeda salsa and Spartina anglica
Source: Arch Microbiol. 2022 Aug 17;204(9):561. doi: 10.1007/s00203-022-03129-9 (PMC9385829; doi:10.1007/s00203-022-03129-9)
Supplement: Supplementary file 1 — Supplementary file1 (DOC 104 KB) [file 203_2022_3129_MOESM1_ESM.doc]

**Supplementary materials for**

**The potential of halophilic-endophytic bacteria for biotite weathering under high-salinity conditions**

Jun Xi^1*^, Kaiqiang Qian^1^, Lidong Shan^1^, Jing Huang^2^, Yanan Yan^2^

**This PDF file includes:**

**Table. S1**

**Table S1.** The detailed information of the rock-weathering halophilic bacteria

| **Strains** | **GenBank accession numbers** | **Fe released (μM)** | **Si released (μM)** | **K released (μM)** | **pH** |
| --- | --- | --- | --- | --- | --- |
| CK |  | 0.25±0.03 | 276.82±5.42 | 123.43±1.43 | 7.43±0.04 |
| SSG1 | JF775411 | 6.98±0.08 | 375.48±9.02 | 266.05±10.08 | 4.94±0.07 |
| SSG3 | JF775426 | 0.46±0.05 | 336.29±11.61 | 331.87±23.1 | 4.94±0.02 |
| SSG4 | JF775413 | 0.45±0.09 | 375.14±11.52 | 319.85±0.15 | 5.00±0.03 |
| SSG7 | JF775427 | 0.75±0.01 | 251.32±0.86 | 180.28±6.06 | 7.01±0.07 |
| SSG9 | JF775443 | 2.4±0.18 | 407.96±16.82 | 169.77±22.92 | 6.81±0.28 |
| SSG10 | JF775444 | 0.88±0.16 | 442.39±31.37 | 231.82±23.9 | 4.98±0.77 |
| SSG11 | JF775428 | 2.24±0.1 | 273.43±2.02 | 220.26±16.61 | 7.51±0.07 |
| SSG12 | JF775445 | 2.28±0.52 | 601.07±20.2 | 198.41±19.73 | 5.24±0.1 |
| SSG13 | JF775412 | 4.94±0.13 | 497.19±67.99 | 185.95±6.38 | 5.26±0.46 |
| SSG15 | JF775414 | 4.37±0.28 | 267.89±4.29 | 200.31±4.57 | 5.12±0.33 |
| SSG17 | JF775446 | 1.57±0.11 | 513.48±49.62 | 235.32±24.71 | 4.9±0.05 |
| SSG20 | JF775429 | 0.55±0.08 | 276.43±5.56 | 214.09±9.67 | 5.3±0.05 |
| SSG22 | JF775430 | 0.55±0.12 | 374.64±4.65 | 289.08±5.84 | 4.8±0.08 |
| SSG25 | JF775431 | 0.44±0.13 | 370.74±2.37 | 279.3±6.18 | 4.66±0.4 |
| SSG34 | JF775447 | 0.65±0.18 | 398.75±23.08 | 288.26±6.73 | 4.95±0.09 |
| SAG36 | JF775415 | 4.11±0.47 | 376.25±5.71 | 248.85±10.33 | 6.05±0.1 |
| SAG37 | JF775416 | 3.58±0.13 | 333.29±26.07 | 220.28±2.28 | 6.38±0.55 |
| SSG39 | JF775417 | 1.22±0.27 | 386.64±31.67 | 205.35±15.54 | 6.27±0.8 |
| SSG41 | JF775448 | 2.04±0.25 | 481.14±18.79 | 240±13.92 | 4.77±0.03 |
| SAG45 | JF775449 | 3.17±0.4 | 229.82±4.19 | 224.95±14.25 | 8.29±0.76 |
| SSY2 | JF775450 | 0.53±0.06 | 307.36±11.55 | 179.13±0.58 | 4.9±0.32 |
| SAY4 | JF775437 | 0.54±0.08 | 336.96±9.55 | 310.39±26.7 | 4.89±0.25 |
| SSY6 | JF775438 | 3.14±0.53 | 282.11±14.39 | 172.21±27.34 | 5.71±0.07 |
| SSY7 | JF775420 | 3.08±0.13 | 454.11±17.32 | 215.23±0.65 | 5.15±0.14 |
| SSY8 | JF775439 | 2.71±0.05 | 474.96±34.7 | 200.08±15.34 | 5.58±0.2 |
| SSY9 | JF775451 | 2.48±0.03 | 259.79±5.76 | 190.92±1.67 | 6.65±1.01 |
| SAY10 | JF775421 | 0.38±0.04 | 453.75±26.52 | 131.2±2.74 | 6.4±0.23 |
| SAY12 | JF775452 | 0.74±0.05 | 324.11±10.56 | 211.08±1.16 | 5.33±0.09 |
| SAY15 | JF775453 | 2.39±0.35 | 268.64±5.92 | 167.05±9.9 | 4.94±0.11 |
| SSY16 | JF775454 | 4.23±0.18 | 379.39±16.01 | 236.03±0.69 | 6.65±0.41 |
| SSY17 | JF775422 | 0.73±0.15 | 586.79±4.55 | 182.67±18.78 | 5.26±0.49 |
| SSY20 | JF775440 | 0.55±0.03 | 557.32±14.9 | 177.11±11.4 | 6.93±0.05 |
| SSY25 | JF775441 | 5.19±0.48 | 337.12±11.93 | 246.05±12.69 | 5.03±0.07 |
| SSY33 | JF775442 | 0.35±0.25 | 270.32±38.94 | 225.26±4.32 | 7.51±0.06 |
| SSY34 | JF775423 | 4.83±1.1 | 250.04±9.85 | 178.28±0.4 | 5.05±0.02 |
| SSY39 | JF775424 | 6.58±0.58 | 328.93±0.3 | 233.05±3.37 | 4.75±0.06 |
| SSY51 | JF775455 | 1.39±0.32 | 395.55±35.68 | 182.62±15.44 | 4.94±0.02 |
| SSY52 | JF775456 | 1.8±0.11 | 561.19±15.68 | 179.65±26.85 | 5.42±0.04 |
| SSY69 | JF775425 | 0.35±0.02 | 386±42.14 | 267.56±10.77 | 5.04±0.1 |
| SAY70 | JF775457 | 2.6±0.16 | 241.46±0.86 | 216.49±4.82 | 7.16±0.15 |
| SAG107 | JF775432 | 0.65±0.05 | 309.52±14.18 | 275.28±15.62 | 4.86±0.08 |
| SAG120 | JF775433 | 0.85±0.05 | 318.83±36.52 | 218.91±2.74 | 4.85±0.01 |
| SAG149 | JF775434 | 1.01±0.18 | 515.96±9.75 | 240.51±5.78 | 5.9±0.02 |
| SAG155 | JF775458 | 1.19±0.48 | 467.46±18.23 | 193.72±1.41 | 4.95±0.01 |
| SAG165 | JF775459 | 1.25±0.11 | 364.98±25.68 | 223.65±8.21 | 5.88±0.23 |
| SAG168 | JF775460 | 0.55±0.13 | 595.04±48.84 | 325±3.52 | 4.9±0.19 |
| SAY169 | JF775435 | 0.56±0.05 | 266.19±9.44 | 386.36±20.31 | 5.09±0.02 |
| SSG170 | JF775461 | 0.95±0.1 | 300.93±15.25 | 247.71±3.77 | 7.11±0.55 |
| SAY81 | JF775436 | 2.26±0.08 | 488.82±14.9 | 251.04±9.48 | 4.64±0.14 |
| SAY92 | JF775462 | 1.87±0.15 | 278.95±9.25 | 195.87±3.59 | 4.92±0.07 |
| SAY106 | JF775418 | 0.55±0.08 | 260.82±0.86 | 291.95±13.13 | 5.19±0.33 |
| SAY107 | JF775463 | 1.58±0.03 | 273.89±1.26 | 417.49±20.96 | 5.07±0.02 |
| SAY108 | JF775464 | 2.64±0.23 | 261.36±6.76 | 177.41±0.11 | 5.12±0.58 |
| SAY109 | JF775465 | 2.77±0.18 | 326.29±33.23 | 202.9±0.91 | 5.07±0.01 |
| SAY120 | JF775466 | 1.42±0.11 | 255.54±14.8 | 279±6.27 | 4.92±0.1 |
| SAY127 | JF775467 | 3.1±0.1 | 373.82±10.05 | 288.19±10.76 | 4.84±0.01 |
| SAY134 | JF775419 | 0.35±0.01 | 300.1±17.78 | 247.41±26.58 | 7.18±0.05 |
